# Cardiovascular computed tomography imaging for coronary artery disease risk: plaque, flow and fat

**DOI:** 10.1136/heartjnl-2021-320265

**Published:** 2022-01-12

**Authors:** Keith M Channon, David E Newby, Edward D Nicol, John Deanfield

**Affiliations:** 1 Department of Cardiovascular Medicine, University of Oxford, Oxford, UK; 2 Centre for Cardiovascular Sciences, University of Edinburgh, Edinburgh, UK; 3 Royal Brompton and Harefield NHS Foundation Trust, London, UK; 4 Departments of Cardiology and Radiology, Centre for Cardiovascular Prevention and Outcomes, University College London, London, UK

**Keywords:** coronary artery disease, risk factors, coronary stenosis, coronary vessels, multidetector computed tomography

## Abstract

Cardiac imaging is central to the diagnosis and risk stratification of coronary artery disease, beyond symptoms and clinical risk factors, by providing objective evidence of myocardial ischaemia and characterisation of coronary artery plaque. CT coronary angiography can detect coronary plaque with high resolution, estimate the degree of functional stenosis and characterise plaque features. However, coronary artery disease risk is also driven by biological processes, such as inflammation, that are not fully reflected by severity of stenosis, myocardial ischaemia or by coronary plaque features. New cardiac CT techniques can assess coronary artery inflammation by imaging perivascular fat, and this may represent an important step forward in identifying the ‘residual risk’ that is not detected by plaque or ischaemia imaging. Coronary artery disease risk assessment that incorporates clinical factors, plaque characteristics and perivascular inflammation offers a more comprehensive individualised approach to quantify and stratify coronary artery disease risk, with potential healthcare benefits for prevention, diagnosis and treatment recommendations. Furthermore, identifying new biomarkers of cardiovascular risk has the potential to refine early-life prevention strategies, before atherosclerosis becomes established.

## Introduction

Cardiac CT (CCT) imaging has transformed the detection, characterisation and stratification of coronary artery disease (CAD) risk in individuals. Historically, evaluation of CAD was guided by symptoms, and crude measures of myocardial ischaemia with limited sensitivity and specificity from exercise ECG (ExECG) and other stress tests. These provided poor diagnostic and prognostic value. Consequently, invasive coronary angiography (ICA) became the gold standard for diagnosis of CAD. However, ICA has major limitations. First, two-dimensional imaging cannot assess haemodynamic consequences of stenoses, in terms of myocardial ischaemia. Second, the ICA ‘lumenogram’ does not image disease in the vessel wall. Intravascular imaging, using ultrasound (IVUS) or optical coherence tomography, reveals that angiographic assessment of the coronary lumen grossly underestimates the presence, nature and extent of coronary artery plaque.

### Development of CCT

CCT imaging for CAD initially quantified coronary artery calcification, as it was readily detected on CT images, and quantified to generate a coronary artery calcium score (CACS) that represented a surrogate marker of the presence and extent of CAD. Large studies with long-term follow-up confirmed the utility of CACS as a predictor of cardiovascular risk in populations. However, the predictive power of CACS in individual patients is limited. Very low or zero CACS is reassuring and clinically valuable, but age and other prevalent risk factors are major drivers of CACS,^1^ such that most middle-aged or older patients in higher cardiovascular risk groups have elevated CACS. Although increasing CACS with time is associated with a higher likelihood of adverse outcomes, major alterations in risk are not necessarily reflected in changes in CACS in an individual patient. A striking illustration of this is that statin treatment increases the CACS, despite substantially reducing cardiovascular risk. This supports the notion that plaque calcification may reflect plaque stability, with non-calcified plaque underlying cardiovascular events and determining individual cardiovascular risk.

Coronary CT angiography (CCTA) has transformed the non-invasive assessment of CAD, enabling visualisation of the coronary lumen, stenoses and plaque features, in three dimensions (3D). These data are now available at low X-ray exposure (often comparable with CACS), and in short acquisition times that reduce the need for breath-holding or beta-blocker treatment to slow the heart rate. Protocol optimisation however does remain heterogeneous between individual centres internationally.^2 3^

The clinical utility of CCTA is supported by many large multicentre studies. In the PROMISE trial, over 10 000 patients presenting with chest pain were randomised to CCTA or an ischaemia test.^4^ CCTA proved more effective than ischaemia testing in identifying patients with significant CAD and more predictive of adverse cardiovascular events. CCTA reduced the number of people undergoing ICA who were subsequently found not to have significant disease. In the SCOT-HEART Study, which evaluated the role of CCTA, 85% of participants underwent ExECG tests prior to randomisation.^5^ While an abnormal ExECG was a strong predictor of the need for coronary revascularisation and cardiovascular risk, CCTA had greater predictive power for determining cardiovascular death or non-fatal myocardial infarction (MI). The limitations of ExECG were highlighted by its low sensitivity for detection of significant CAD (~0.40).

Current non-invasive imaging strategies to quantify cardiovascular risk using ischaemia testing also have limited value, as shown in the recent ISCHEMIA trial.^6^ The objective of the ISCHEMIA trial was to compare optimal medical therapy (OMT) with coronary revascularisation in >4000 patients with positive ischaemia tests. OMT was as effective as revascularisation for reduction of overall cardiovascular events. The trial mandated that all patients underwent CCTA prior to randomisation to exclude left main stem disease, and exclude false positive functional tests. Subanalysis of the ISCHEMIA trial indicated that CCTA was superior to ischaemia testing for predicting adverse events. Indeed, the severity of ischaemia did not predict mortality, and the association between ischaemia severity and non-fatal MI was lost when adjusted for extent of CAD on CCTA.^7^

The shift away from ischaemia testing is also supported by the SCOT-HEART trial that tested the clinical impact of early CCTA in patients with chest pain, compared with routine clinical care.^8^ CCTA reduced major adverse cardiovascular events over 5 years, without an overall increase in rates of coronary revascularisation for ischaemia, which was only higher in the first year after CCTA. The benefit was associated with a significant increase in the use of optimal medical therapy in patients undergoing CCTA indicating that targeted optimisation of medical therapy in those with significant CAD, irrespective of detectable ischaemia, significantly reduced the likelihood of future adverse cardiovascular events. Thus, ischaemia testing is most useful in assessing symptoms and their relationship to the presence of coronary stenoses, rather than predicting cardiovascular risk.

### Current status of CCT in cardiovascular diagnosis and risk assessment

As a result of these seminal trials, CCTA is used increasingly as a first-line test for patients presenting with chest pain, for diagnosis and to guide management strategy.^9^ Over 20 million patients present with chest pain every year in the USA (3% of primary care visits, 5% of emergency department visits) with >5 million CCTAs performed in Organisation for Economic Co-operation and Development countries. It is estimated that by 2025, 10%–15% of all CT scans performed globally will be CCTAs, and CT hardware manufacturers are now producing CT machines that focus primarily on performing CCTA, in order to make this technology more accessible and cost-effective in healthcare systems.

In 2010, the UK’s National Institute for Health and Clinical Excellence (NICE) recommended that CCTA should be the first-line test for people with recent onset chest pain (NICE CG95). The European Society of Cardiology guidelines, published in August 2019, upgraded CCTA to a first-line investigation with the highest level of evidence (class 1), as the initial test for patients with a low to moderate clinical likelihood of CAD, representing the majority of patients presenting with chest pain. The 2021 US guidelines also reflect the increased utility of CCTA, particularly in younger people.^10^

CCTA generates important additional information, beyond the presence and extent of CAD (as assessed by luminal narrowing and artery wall plaque features) that has actionable clinical importance for patients. The functional significance of stenoses can be calculated using computational flow dynamic or machine learning techniques to derive FFR_CT_, with some studies showing a modest reduction in the need for ICA and intracoronary pressure wire studies.^11–13^ However, in the FORECAST trial^14^, patients who had a CCTA as the first-line test received no additional benefit from FFR_CT_ or reduction in subsequent ICA, and incurred 20% higher healthcare costs.^15^ In contrast, the wider use of CCTA in patients with possible CAD is cost-effective and does not drive an overall increase in ICA rates.^16^

CCTA can quantify the extent, distribution and characteristics of coronary plaques, but even this is not sufficient for optimal risk prediction in individuals. It is well known that most acute MIs occur secondary to occlusion in vessels with minor coronary plaque disease that erodes or ruptures. This relates to the biology of the underlying coronary plaque, particularly inflammation. In the PROMISE trial, 54% of adverse events occurred in patients without significant stenoses, whereas patients with significant stenoses accounted for only 12% of the population undergoing CCTA.^4^ Thus, more than half of the aggregate risk of adverse cardiovascular events is not identified by coronary stenoses in people who undergo CCTA. This limitation is a driver of ‘residual risk’ that results in adverse cardiovascular outcomes, despite efforts to manage cardiovascular disease (CVD) according to current recommendations.

**Figure 1:**
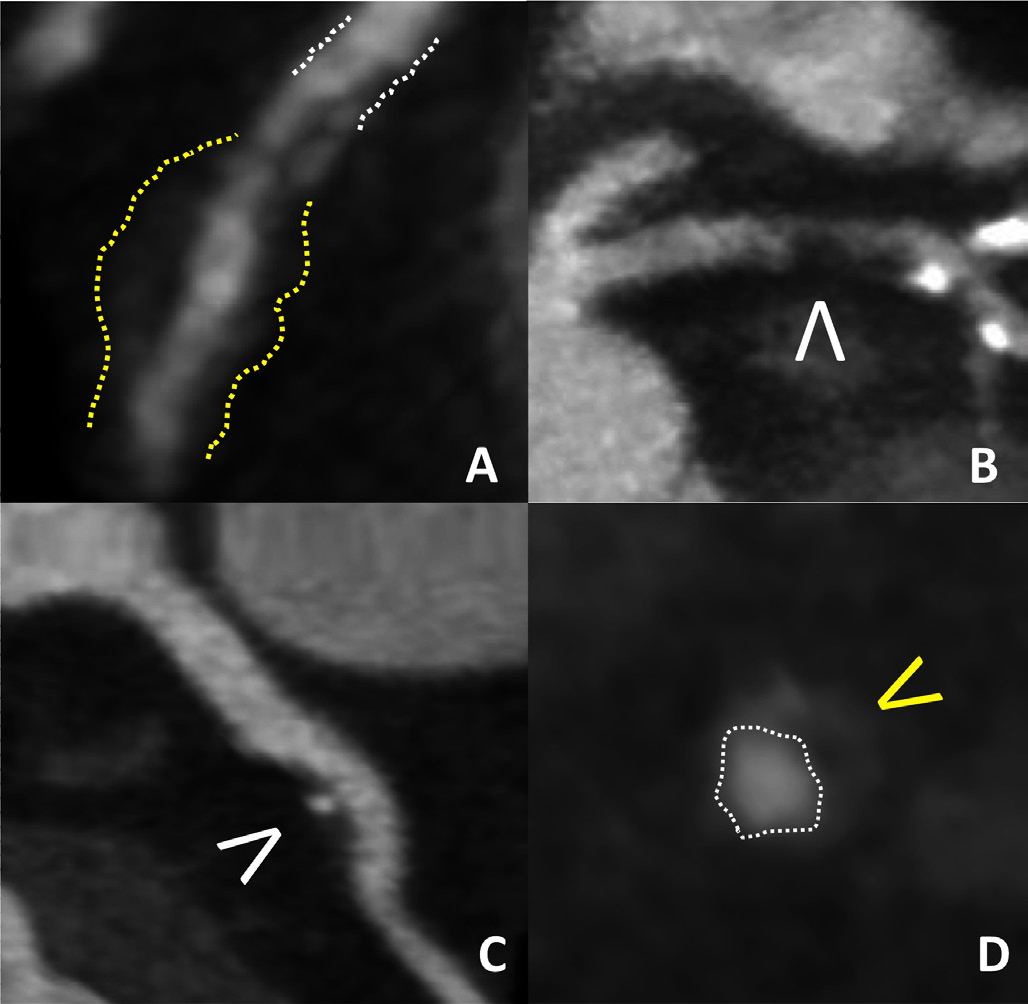
High-risk plaque features on CCTA. (A) Positive (Glagov) remodelling of plaque (yellow lines) maintains the coronary lumen (white lines) by outwardly remodelling atheromatous wall of the vessel. (B) Low-density non-calcified plaque (<30 HU) (white arrowhead). (C) Spotty calcification (white arrowhead. (D) Napkin ring sign (lumen—white line; plaque—yellow arrowhead). CCTA, coronary CT angiography.

CCTA can identify patients with plaque characteristics associated with high risk, such as low-attenuation plaque, napkin ring sign, positive remodelling and spotty plaque calcification (figure 1); however, these provide only modest incremental information in individual patients. The predictive value of high-risk plaque (HRP) features was studied in both the PROMISE and SCOT-HEART trials. In the PROMISE Study, ~700 of 4400 (~15%) patients were found to have HRP on their CCTA^17^ and HRP was associated with more adverse cardiovascular events, (6.4% vs 2.4%; HR 2.73, 95% CI 1.89 to 3.93), although the major adverse cardiovascular events (MACE) endpoint included revascularisation which may not reflect the additional value of HRP above and beyond stenosis severity. Nevertheless, most patients with HRP did not have cardiovascular events, whereas many patients without HRP did, indicating the limited predictive value of HRP on CCTA. Indeed, of the 1019 HRPs identified on CCTA, only 24 subsequent non-fatal MIs occurred, demonstrating that the absolute risk of a cardiovascular event in relation to a single plaque, identified at a single time point, is extremely low.

This observation is consistent with other CCTA and IVUS studies. In the SCOT-HEART trial, 1376 HRP features on CCTA were detected in 608 of 1769 participants.^18^ The likelihood of an adverse cardiac event during follow-up was increased in the subjects with HRP features, but the absolute increase in risk was very small (4.1% with HRP vs 1.4% without HRP). Importantly, more than one-third of the events occurred in subjects without HRP features. A more detailed analysis identified low-attenuation non-calcified plaque burden as the most specific HRP feature predictive of adverse events.^19^

The PROSPECT Study used virtual histology-IVUS (VH-IVUS) to image coronary plaque in all three major coronary arteries of patients undergoing ICA following an acute coronary syndrome (ACS).^20^ HRP features on VH-IVUS (thin cap fibroatheromas) were identified in 594 plaques, but only six patients subsequently had a subsequent MI over 3.4 years. The PROSPECT II Study combined VH-IVUS with intracoronary near infrared spectroscopy to evaluate lipid-rich plaques.^21^ Of more than 3600 non-culprit lesions in approximately 900 patients with an ACS, large plaque burden and lipid-rich plaques were associated with increased likelihood of events. However, over 3.7 years of follow-up, only 78 non-culprit lesions caused adverse events. These and other studies emphasise that the relationship between the presence of ‘vulnerable’ plaques, rupture or erosion at the site of any individual plaque and clinical events is very weak. Recent discoveries highlight the importance of cellular inflammatory mechanisms in the vascular wall as drivers of disease progression and risk of events.^22^

CCTA shows great promise for early risk assessment in otherwise healthy individuals^23 24^; however, to achieve greater accuracy in cardiovascular risk prediction, non-invasive assessment of CAD needs to identify more than coronary plaques, the degree of stenosis, HRP features and the presence of functionally significant lesions by FFR_CT_. To improve prediction of future adverse cardiovascular events needs, CCTA data sets need to identify local biological processes that drive disease and events, such as inflammation. Coronary artery inflammation is a major factor in CAD progression, and a key determinant of high-risk plaques that drive adverse clinical events, in addition to the contributions of stenosis, flow limitation or adverse plaque features.^22^

### Adipose tissue imaging to identify cardiovascular inflammation

Imaging perivascular adipose tissue (PVAT) around the coronary arteries has emerged as a promising technique to image inflammation in the coronary artery wall. A key recent discovery is that PVAT ‘senses’ the presence of inflammation in the wall of the coronary artery. These signals transduce changes in PVAT differentiation, leading to smaller, less lipid-rich adipocytes, corresponding to transcriptomic changes in adipocyte gene expression, greater inflammatory cell infiltration and higher tissue water content.^25^ These changes modify tissue attenuation values in a 3D distribution around the coronary artery that can be detected using CCTA, enabling derivation of new imaging biomarkers from PVAT attenuation as a marker of coronary artery inflammation.^25^ These changes can be measured prospectively or retrospectively, making it a readily accessible technique. Simple measures of PVAT attenuation require corrections for anatomical, technical factors, and patient and clinical variables. However, the validity of measuring changes in pericoronary fat attenuation has been reproduced in research studies performed using similar techniques in different patient groups.^26–28^ A CE-marked medical device, CaRi-Heart, is now available to quantify the Fat Attenuation Index (FAI)-Score from routine CCTAs, providing a per-vessel readout of coronary inflammation, and integrates FAI-Score with clinical factors to generate a personalised risk estimate.^29^

The CRISP-CT Study demonstrated the clinical value of PVAT imaging using a derivation cohort of 1872 patients (from Erlangen, Germany), and a validation cohort of 2040 patients (from Cleveland Clinic, USA) who had undergone CCTA and were followed up for up to a median of 9 years.^30^ Following correction for demographic and clinical risk factors, and for the presence of coronary plaque identified in the CCTA, elevated PVAT attenuation (greater than the calculated cut-off of −70.1 HU), on the index CCTA, conferred a relative risk of subsequent all-cause mortality of ~3-fold, a risk of cardiovascular death of ~7-fold and a risk of acute MI of ~5-fold. In patients with HRP features on CCTA, perivascular fat attenuation is increased in those that have increased ^18^F sodium fluoride uptake on positron emission tomography imaging, a marker of plaque inflammation.^31^ Furthermore, the abnormal PVAT attenuation associated with coronary inflammation appears to be dynamic. For example, PVAT attenuation adjacent to lesions that have been treated by percutaneous coronary intervention in patients with acute MI rapidly normalises after the acute events. PVAT attenuation also normalises after initiation of statin treatment,^30^ or after anti-inflammatory treatments in conditions such as psoriasis.^32^

**Figure 2:**
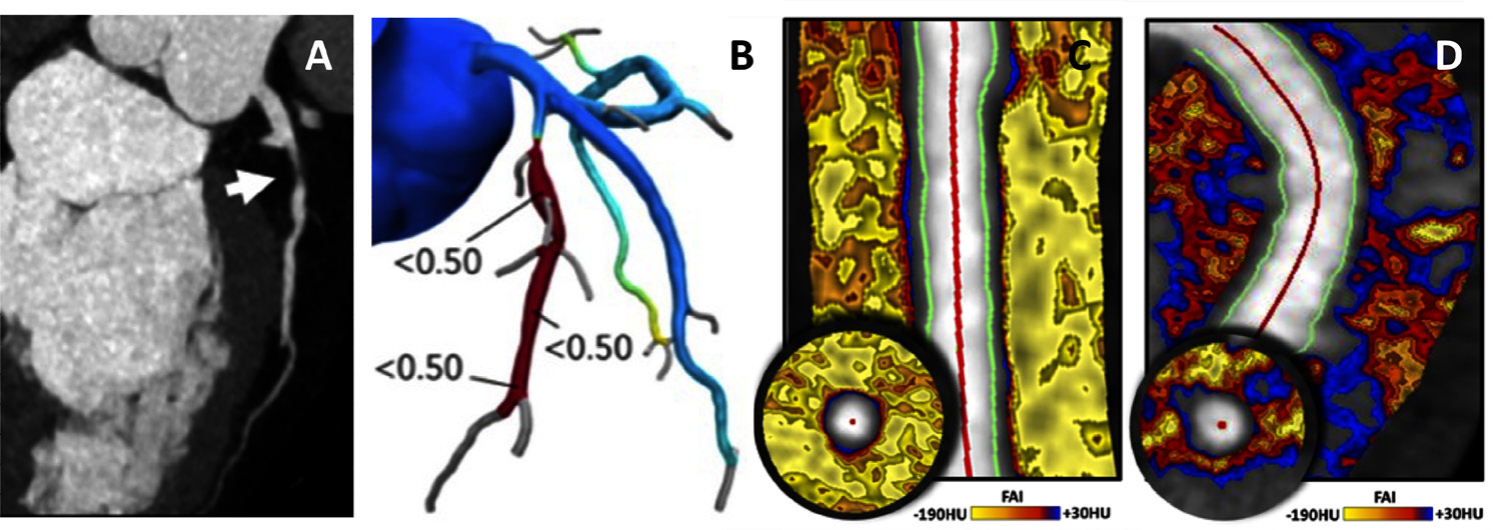
CAD characteristics evaluated by CCTA. (A) Non-calcified, causing a significant luminal narrowing (70%–99%), with (B) computational CT-fractional flow reserve (FFR) indicating a significant FFR across the lesion. (C) Low Fat Attenuation Index (FAI) of the perivascular adipose tissue (predominant yellow shading), whereas an angiographically similar coronary segment shows marked areas of low-attenuation perivascular adipose tissue (red-blue), indicating inflammation and a high risk of future cardiovascular events, despite no apparent luminal atheroma. CAD, coronary artery disease; CCTA, coronary CT angiography.

**Figure 3:**
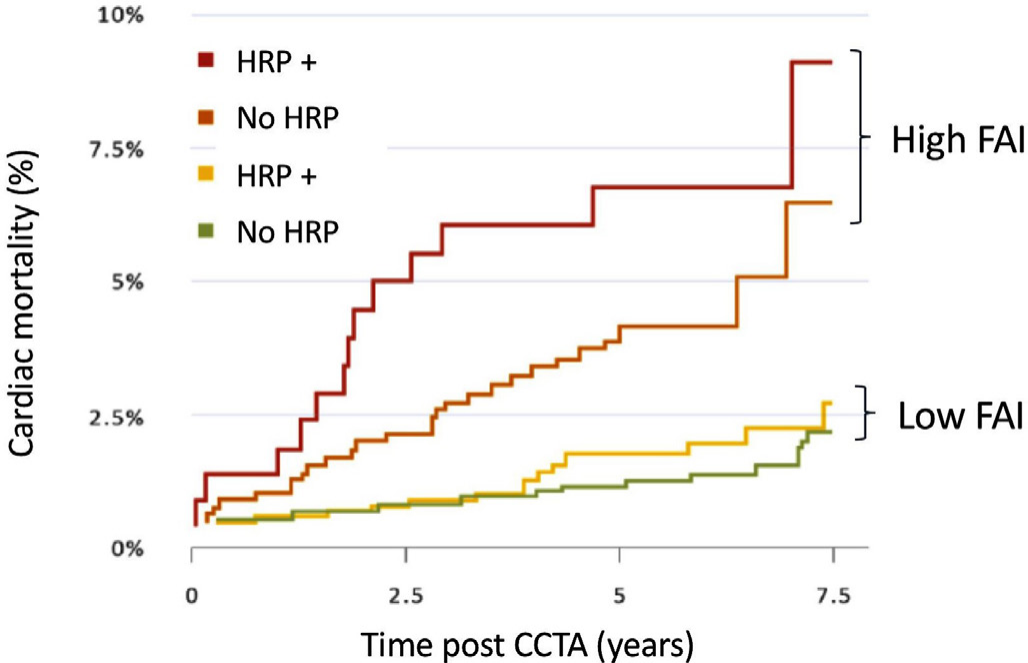
Detection of coronary inflammation using perivascular Fat Attenuation Index (FAI) and prediction of cardiovascular risk. Kaplan-Meier curves with adjusted HRs for patients with either high or low FAI quantified around the proximal right coronary artery (high FAI >−70.1 HU, low FAI <−70.1 HU), with or without high-risk plaque (HRP) features identified on the CCTA. Figure adapted from Oikonomou *et a*l.^33^ CCTA, coronary CT angiography.

Combining coronary PVAT inflammation from CCTA with HRP features may provide additional insights into the relative importance of ‘biological’ versus ‘structural’ readouts for cardiovascular risk prediction and for understanding CAD pathogenesis (figure 2). A recent analysis of the CRISP-CT Study categorised 3912 patients as having at least one HRP feature, or not, or having high or low FAI, relative to the established cut-off of −70.1 HU.^33^ FAI remained a highly significant predictor of adverse cardiovascular outcomes, even in patients without HRP features. Furthermore, the magnitude of risk flagged by high FAI, even in patients without HRP features, was greater than the risk conferred by the presence of HRP features in patients with low FAI. The aggregate risk of high FAI and HRP features was greatly increased—by approximately 7.3-fold (96% CI 3.4-fold to 15.8-fold)—compared with the lowest risk group. These findings indicate that high FAI, in the context of patients with HRP features, identifies a small but very high-risk subgroup. However, the novel predictive power of FAI is most evident in the large majority of patients who do not have HRP features on CCTA, but who have a high and clinically actionable increase in cardiovascular risk (figure 3). This observation reflects the importance of otherwise undetectable coronary inflammation in driving cardiovascular events in apparently ‘low-risk’ individuals, and highlights the additional predictive value of coronary artery inflammation, and the complex cellular processes driving atherosclerotic risk in the vascular wall, compared with markers of systemic inflammation such as high sensitivity C-reactive protein.^22^

The evaluation of FAI from the pericoronary adipose tissue imaged by CCTA offers the potential to derive other biomarkers from CCTA that reflect molecular changes related to cardiovascular risk. The ‘radiotranscriptomic’ approach, used to derive FAI from CT images of adipose tissue biopsies that underwent transcriptomic analysis of adipose tissue gene expression,^25^ has now been extended to derive new signatures that reflect fibrosis and vascularity.^34^ For example, new radiomic features of pericoronary adipose tissue texture were related to fibrosis (*COL1A1* expression) and vascularity (*CD31* expression). These features significantly improved prediction of adverse cardiovascular events in 1575 participants in the SCOT-HEART trial, beyond traditional risk stratification (including clinical risk factors, CACS, coronary stenosis and HRP features).^34^

FAI provides a powerful, convenient and clinically applicable tool to stratify cardiovascular risk based on CCT scans performed routinely in clinical practice. It has the potential to reclassify CVD risk and treatment allocation, leading to earlier and improved CVD diagnosis and treatment, which may reduce CVD mortality and morbidity, improve cost-effectiveness and reduce the economic burden of CVD.

A recent algorithm demonstrates how incorporation of FAI analysis in patients undergoing CCTA could improve risk stratification and clinical management pathways.^35^ The majority of people who currently undergo CCTA are labelled as ‘low risk’, but many go on to have coronary events. An abnormal FAI identified on the index CCTA could reclassify patients as ‘high risk’ and offered more personalised preventive management.^2^ Conversely, a low FAI in patients labelled as ‘normal’ by conventional CCTA could provide additional reassurance. Patients with significant CAD on CCTA should already receive conventional medical treatment, but cardiovascular events in this group continue to occur, reflecting ‘residual risk’.^35^ FAI could help to refine the management of patients shown to have significant CAD on CCTA, even after the use of conventional medical therapy. An elevated FAI may identify patients who remain at high risk, and enable more cost-effective use of novel anti-inflammatory therapies.

The ability to detect coronary artery inflammation, non-invasively, opens up the potential to understand the drivers of early CAD to prevent atherosclerotic plaque progression, before it confers significant cardiovascular risk. CAD begins very early in life, with inflammation driven by exposure to potentially modifiable risk factors. While the absolute risk of major cardiovascular events in younger people is low, CAD progresses silently over the life course, for many years before cardiovascular events occur. Many individuals in the population have an accelerated trajectory for the development of CAD, and could benefit from early-life identification and more proactive implementation of prevention measures. These are currently limited to generic health advice on smoking, cholesterol, blood pressure and lifestyle. Coronary artery inflammation is now recognised as a major factor in early disease pathogenesis, and is driven by classic cardiovascular risk factors and influences such as obesity, systemic inflammation and genetic susceptibility. Non-invasive detection of coronary artery inflammation is feasible by pericoronary adipose tissue imaging, even in young patients, and the significance of this will be an important focus for future studies. These will improve understanding of the pathophysiology of early atherosclerosis and guide timing and choice of early risk factor therapy as part of a personalised prevention approach. A focus on prevention is central to the current National Health Service Long Term Plan.

## Conclusions

**Figure 4:**
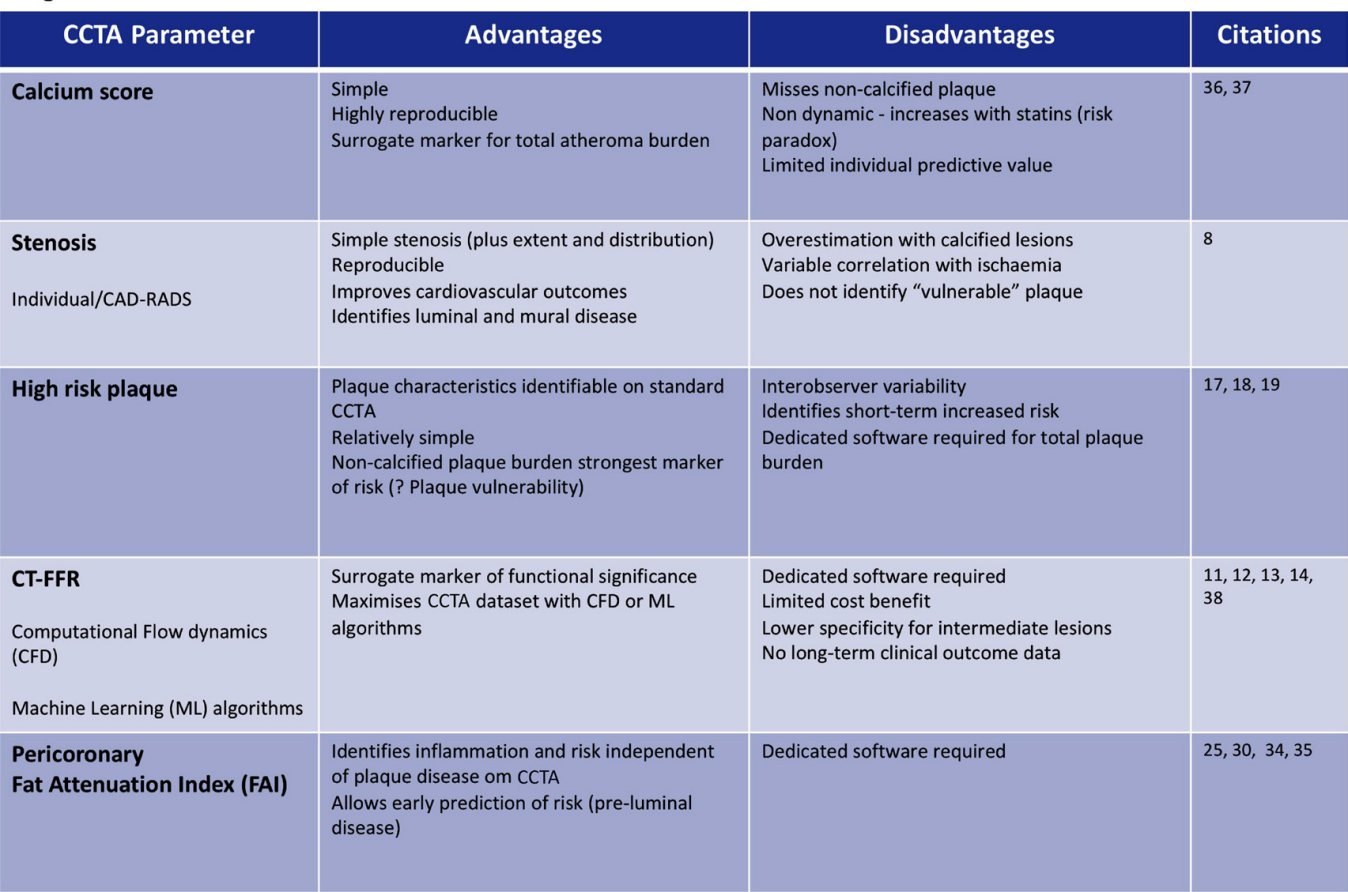
Table showing comparative characteristics of CT-derived parameters of coronary plaque, coronary stenosis and perivascular adipose tissue. CAD, coronary artery disease; CCTA, coronary CT angiography.

CCTA has emerged as the preferred non-invasive modality for the study of chest pain due to possible underlying CAD, and for the assessment of cardiovascular risk. Technical advances and realignment of patient pathways make CCTA a first-line investigation for assessment of CAD (figure 4). CCTA can identify plaque, assess stenosis, infer the presence of ischaemia from functional modelling and identify plaque features that are associated with high risk of future clinical events. However, even these advances are not sufficiently sensitive to comprehensively stratify cardiovascular risk. Many patients with ‘reassuring’ CCTA findings, that currently do not predict high risk, subsequently suffer MACE, reflecting the ‘residual risk’ that remains invisible to current imaging techniques. Perivascular fat attenuation is a novel biomarker of coronary artery inflammation that can be evaluated from routine CCTA. It has the potential to predict coronary risk, above and beyond plaque features or stenosis, and is able to reclassify a substantial proportion of patients. This new technology has important implications for cardiovascular risk stratification, and for new approaches to cardiovascular risk prevention, both in individuals and in populations.
